# Addressing *barriers in comprehensiveness, accessibility, reusability, interoperability and reproducibility of computational models in systems biology*

**DOI:** 10.1093/bib/bbac212

**Published:** 2022-06-08

**Authors:** Anna Niarakis, Dagmar Waltemath, James Glazier, Falk Schreiber, Sarah M Keating, David Nickerson, Claudine Chaouiya, Anne Siegel, Vincent Noël, Henning Hermjakob, Tomáš Helikar, Sylvain Soliman, Laurence Calzone

**Affiliations:** Université Paris-Saclay, Laboratoire Européen de Recherche pour la Polyarthrite rhumatoïde - Genhotel, Univ Evry, Evry, France; Lifeware Group, Inria, Saclay-île de France, 91120 Palaiseau, France; Department of Medical Informatics, University Medicine Greifswald, Greifswald, Germany; Biocomplexity Institute and Department of Intelligent Systems Engineering, Indiana University, Bloomington, IN, USA; Department of Computer and Information Science, University of Konstanz, Konstanz, Germany; Faculty of Information Technology, Monash University, Clayton, Australia; University College London, London, United Kingdom; Auckland Bioengineering Institute, University of Auckland, Auckland, New Zealand; Aix Marseille Univ, CNRS, I2M, Marseille, France; Univ Rennes, CNRS, Inria - IRISA lab. Rennes; Institut Curie, PSL Research University, Paris, France; INSERM, U900, Paris, France; MINES ParisTech, PSL Research University, CBIO-Centre for Computational Biology, Paris, France; EMBL-European Bioinformatics Institute, Wellcome Genome Campus, Cambridge, UK; Department of Biochemistry, University of Nebraska-Lincoln, Lincoln, NE, 68588, USA; Lifeware Group, Inria, Saclay-île de France, 91120 Palaiseau, France; Institut Curie, PSL Research University, Paris, France; INSERM, U900, Paris, France; MINES ParisTech, PSL Research University, CBIO-Centre for Computational Biology, Paris, France

**Keywords:** computational modelling, model annotations, reproducibility, systems biology, community standards

## Abstract

Computational models are often employed in systems biology to study the dynamic behaviours of complex systems. With the rise in the number of computational models, finding ways to improve the reusability of these models and their ability to reproduce virtual experiments becomes critical. Correct and effective model annotation in community-supported and standardised formats is necessary for this improvement. Here, we present recent efforts toward a common framework for annotated, accessible, reproducible and interoperable computational models in biology, and discuss key challenges of the field.

## Introduction

**Figure 1:**
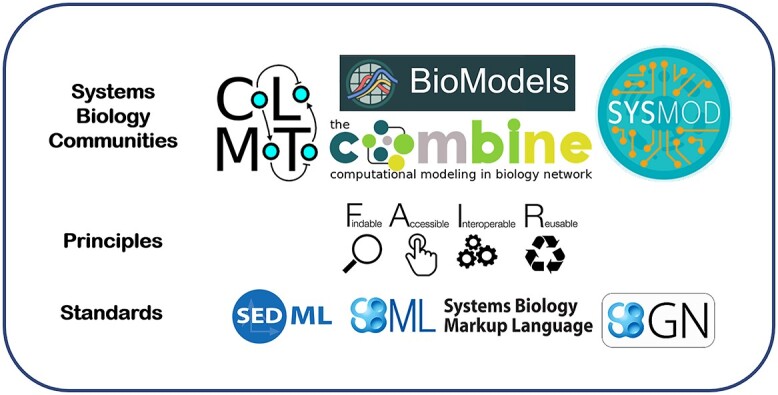
The systems biology communities represented in the review, along with the main principles and community standards discussed.

Scientists from different systems biology fields have long been developing community-driven guidelines and best practices for annotation, interoperability and reusability of computational models in biology. However, the parallel work, grounded on shared needs and similar aims, of separate communities creates a need for exchange and alignment of the different efforts to harmonise best practices. Hence, members of the Consortium for Logical Models and Tools (CoLoMoTo, http://colomoto.org) and the Computational Modelling of Biological Systems community of the International Society for Computational Biology (SysMod, https://sysmod.info/) organised a workshop to discuss community-driven guidelines and efforts for the curation and annotation of computational models during [BC]2 2021. The workshop grew from a previous edition organised during [BC]2 2019 focused on logical modelling [1]. The second edition brought together scientists with various research backgrounds and from different working groups such as BioModels [2], a central repository of mathematical models of biological/biomedical processes; the Computational Modelling in Biology Network initiative (COMBINE) [3]; CoLoMoTo, [4]; SysMod, [5]; the Systems Biology Graphical Notation (SBGN) project [6]; the systems biology markup language (SBML) [7] and simulation experiment description markup language (SED-ML) [8], to exchange and expand on several key topics of common interest (Figure 1).

**Figure 2:**
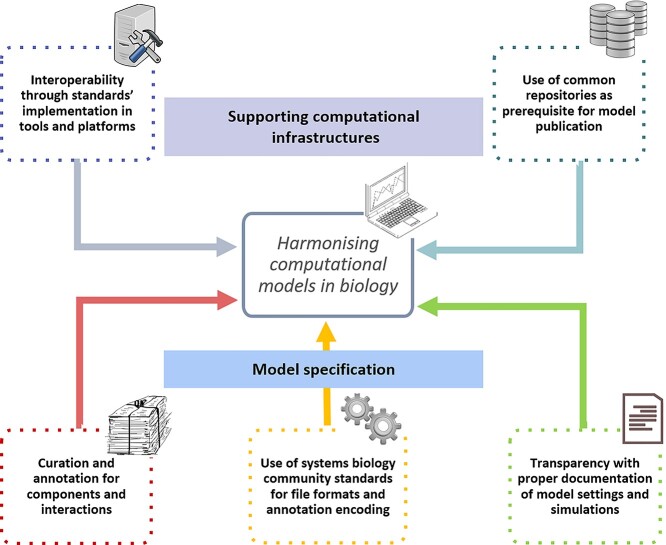
Overview of key needs to harmonise computational models in biology. (Science vectors from https://freesvg.org/science-icons-set-vector-image).

While the modelling approaches across these communities differ, several critical points are shared, such as (i) the importance of annotations for reproducibility, (ii) the use of community standards for exchange and annotation encoding, (iii) the need to implement standards in tools and platforms to boost reusability and interoperability, (iv) the importance of transparency of modelling frameworks in publications and (v) the use of shared repositories to enhance model accessibility (Figure 2). We use the term annotation to describe ‘a computer-accessible metadata item that captures, entirely or in part, the meaning of a model, model component or data element’. We borrow this definition from [9] which is in accordance with its use in [1]. We discuss the identified needs in the following sections.

## Model curation and annotation, and the use of community standards

Computational models have long been used to describe complex systems in biology. Their abstract nature and their ability to reproduce dynamic behaviours make them very powerful tools for hypothesis testing and *in silico* predictions. Furthermore, computational models are built based on a wealth of biological data, both low and high throughput, and their integration with prior, empirical knowledge. The majority of these models are based on small-scale experimental observations integrated with large-scale omic data, and a number of formalisms have been developed over the years to address different needs and questions regarding the biological systems under study [10–12].

The standardised representation of biological mechanisms is important for both static and dynamic systems biology models. Efforts to formalise process description (PD) diagrams using SBGN schemes [6] have been made within the Disease Maps project (https://disease-maps.org/) [13] and, more recently, within the COVID-19 Disease Map community [14]. Tools to automate the translation of Disease Maps into executable Boolean models, such as the CellDesigner as SBML-Qual (CaSQ) tool, have eased the creation of models in the SBML-qual format [15, 16]. This initiative has demonstrated the absolute need for coordinated efforts among different systems biology communities to establish best practices for model curation and annotation [1]. The use of standards, especially SBGN [6], reduces uncertainty and disambiguates knowledge representation. SBGN includes three complementary and orthogonal visual languages which represent alternative projections of the underlying biological system: (a) the SBGN PD language [17], which shows the sequence of interactions between biochemical entities on a mechanistic level; (b) the SBGN Entity Relationship language [18] that represents the relationships in which an entity can participate regardless of the sequential order of these events and also shows information on a mechanistic level and (c) the SBGN Activity Flow language [19] that depicts influences between entities in a network on a more abstract level.

Tools are needed to implement the three SBGN languages in a straightforward, user-friendly way. An example is SBGN-ED [20], an editor capable of handling all three SBGN languages. SBGN-ED allows the user to check for correctness using rule-based methods, combine SBGN bricks to build networks, translate diagrams between formats [21], apply automated layouts to improve the readability of maps [22–24], and perform data integration tasks such as converting high-throughput (omic) data into pathway maps [25].

Such pathway maps can also be imported in Cytoscape [26] and analysed as graphs for their topology, revealing important structural properties such as various centrality measurements, in and out degrees, and network connectivity.

An important aspect for model reusability is the capability to merge and combine existing models. The use of standards facilitates model integration as it minimises the assumptions needed to combine different constructs. CellDesigner [27] is a structured diagram editor for drawing and modelling biochemical networks, which supports SBGN standards and implements model merging via a dedicated plugin, thus giving the opportunity to create merged models, combining two or more diagrams. In the same vein, SemGen [28] is a tool developed recently that is able to synthesise models encoded in various formats, including SBML [7] and CellML [29]. The tool relies on semantic annotations to capture the underlying biological and physical meanings of the model entities and processes. MultiState Model Builder (MSMB) is another effort to create a flexible editor for compact biochemical models [30, 31]. MSMB supports multistate models created using different modelling styles and is based on Java and COmplex PAthway SImulator (COPASI) [32] APIs. Simulink, a MATrix LABoratory (MATLAB) toolbox, is a block-diagram environment for multidomain simulation and model-based design. It includes a merge functionality allowing the merger of two versions of a model [33].

Recent developments of SBML have enabled the support of multiple modelling methodologies [7]. SBML is a powerful language whose syntax supports annotations and covers various modelling frameworks, including rule-based, logical, spatial, kinetic methodologies and multiple types of analysis, including time-course simulations, parameter estimation, sensitivity analysis, flux balance analysis and visualization. However, one key field challenge that remains to be addressed is to capture both the mathematics and the semantics that define models and simulations in an implementation-independent manner.

## Interoperability through standards’ implementation in tools and platforms

The findability, accessibility, interoperability and reusability (FAIR) principles for data stewardship were published in 2016 and intended to provide guidelines and enhance the above features in research objects related to computational modelling [34]. Since then, the FAIR principles have been applied to research fields ranging from biology to physics to health sciences. Bioinformatics and biology as a whole have been early adopters of FAIR, and data management systems like the FAIRDOMHub [35] aid the creation of fair scientific data. In addition, the biomedical and health domains recently started to investigate FAIRification approaches [36–39].

Standards, shared repositories and community-driven tool developments improve the reproducibility of scientific outcomes [40, 41]. The appropriate reuse of models requires easy access and a certain comprehensiveness of relevant models, which can be achieved via the annotation and/or documentation, or comments in a modelling script. Findability and comprehensiveness require proper archiving and sharing of a mathematical model encoded in a standard format, accompanied by thorough descriptions of the model and its application in simulation experiments. Adding structured annotations to the model archive [9, 42] and sharing them via suitable repositories greatly facilitate the search for models and reuse by different software tools [43]. An essential aspect of reusability is model versioning that allows for tracking model provenance correctly and properly crediting the source model. Initiatives such as those included in the *Physiome* journal (https://journal.physiomeproject.org) are encouraging such practices by incorporating them into traditional academic credit metrics.

An excellent example of formalised model reuse and merge is described in [44] in which the authors combined three different models of rat cardiomyocyte function (an electrophysiology model [45], a dynamic model of calcium intake and release [46] and a quantitative, mechanistic model [47]) to build an integrative cell model for cardiac modelling. This work highlighted the added value of integrative models and pointed to the challenges associated with such endeavours.

Regarding graphical models, the integration of the mastocyte activation PD map built in CellDesigner and published in [48] into the REACTOME pathway knowledge base [49], as part of the Fc epsilon receptor signalling pathway (https://reactome.org/content/detail/R-HSA-2454202), is a nice example of model reuse with proper attribution of credits to all contributors.

Combining complementary modelling tools strengthens model analysis, making interoperability essential for modelling pipelines. A combined pipeline for model building and analysis is illustrated in a recent publication in which researchers built a logical model of the epithelial-to-mesenchymal transition (EMT) cellular network to assess how selected microenvironmental signals control cancer-associated phenotypes along the EMT continuum [50]. Their pipeline uses the Gene Interaction Network Simulation (GINsim) tool (http://ginsim.org) [51] to build the model and identify steady states, two R packages, FactoMineR [52] and factoextra (https://rpkgs.datanovia.com/factoextra), to cluster the corresponding phenotypes and BoolSim [53] to confirm the absence of cyclical attractors. They also used the model checker New Symbolic Model Verifier - Action Restricted Computation Tree Logic (NuSMV-ARCT) [54] to perform model checking analysis and finally MaBoSS for stochastic simulations [55]. While not all tools use standard formats, they can produce compatible, intermediate files that can be imported and analysed further. However, adopting standardised practices through shared libraries and open-source code on how tools handle model building and analysis could significantly accelerate community-driven software updates and the development of multifunctional and seamless analytical pipelines. To support this adoption, the CoLoMoTo interactive notebook [56] is a community-driven effort to improve reproducibility and reusability within the subdomain of logical models and software tools. Combined with suggestions for data retrieval [57], data integration [58] and proper annotation [1] it provides a fairly complete suggestion for best practices for building logical models in biology.

Similarly, the Stimulating Peripheral Activity to Relieve Conditions (SPARC) initiative (https://sparc.science/) offers a dedicated portal for data, knowledge, computational modelling and spatial mapping for the peripheral nervous system, enhancing findability via semantic search and interface, and reuse of resources among scientists interested in the nervous system [59].

A recent community-driven initiative, which aims at providing an integrative environment for model simulations, is BioSimulators (https://biosimulators.org/), a central registry of various simulation tools along with programmatic interfaces to different tool versions. BioSimulators relies heavily on systems biology community standards, such as CellML, SBML, SED-ML and the COMBINE archive format, as well as validation tools to ensure proper implementation and use of these standards. The registry also offers recommendation services, in an effort to help modellers select the best way to share, reproduce and combine simulations [60].

The careful specification of simulation settings is also essential to ensure reproducibility of modelling results. The SED-ML (https://sed-ml.org/) community focuses on developing a standard to cater to this need. The SED-ML format includes information required by the minimum information about a simulation experiment (MIASE, http://co.mbine.org/standards/miase) to ensure the reproducibility of computational simulations. In addition, SED-ML covers the description of time-course simulations related to quantitative, kinetic models. A working group is currently working on a much-needed SED-ML extension for logical models that would better describe the simulation settings for discrete logic-based models.

In summary, extensive, standardised and meaningful annotations and detailed references to source models and data, and transparency throughout the model-building steps make virtual experiments accessible to a broader audience and encourage model reusability. In addition, standardised descriptions of simulation settings improve the reproducibility of *in silico* experiments.

The web-based platform Cell Collective was developed to make computational modelling accessible to a broader panel of users (from students to experts) [61]. Cell Collective can be used to build large-scale models of various biological processes and simulate/analyse them. It includes a knowledge base for information about individual biological processes, such as identifying direct protein–protein interactions. The tool also includes a reference section where users can enter references using PubMed IDs. Finally, the tool provides a large model repository of Boolean and, recently, constraint-based models, and includes analytical methods for both modelling approaches. With similar goals, another interface, WebMaBoSS [62], was developed for simulating models with the Markovian Boolean stochastic simulator (MaBoSS) tool. The MaBoSS software is based on stochastic simulations of Boolean networks [55]: transition rates are associated with each node of the model, and probabilities for the states of the model are computed over time. In addition, the framework was expanded with an easy-to-use web interface. New models can be loaded from local files or imported directly from existing databases, including BioModels (https://www.ebi.ac.uk/biomodels/), Cell Collective (https://cellcollective.org/) and GINsim (http://ginsim.org/models_repository) repositories. Models are then stored in a personal database and modified, keeping track of all versions. This new interface comes with an update of MaBoSS, which improves its compatibility with community formats by adding native support of SBML-qual (16) and BoolNet [63] model representations.

## Transparency through proper documentation across modelling frameworks

The need for curation guidelines and standard formats for archiving information also applies to metabolic models. Genome-scale metabolic models describe an organism’s metabolism and need to be integrated when studying microbial communities at the metabolic level. Building comprehensive models requires integrating all relevant information from multiple biological databases with different data types. Data integration is not straightforward, though, and choices of data and analytical tools need to be made. Furthermore, for models to be fully exploited by the community, reconstruction steps must be well documented and described as metadata in a standardised workflow language. These metadata allow other researchers to reproduce and improve models or reuse models as blueprints to create new ones. Recent efforts include the dedicated *padmet* format to generate customised metabolic models through transparent reproducible pipelines [64]. Moreover, the automatic generation of local wikis and query-based interfaces facilitates the exploration of models and metadata [65].

The same issues apply to the construction of multiscale models. Currently, there is limited interoperability of virtual-tissue simulation environments as they often handle one methodology per scale. In addition, standards for describing cellular and multicellular experiments and models are lacking. There is a clear need to develop cross-scale integration languages and ontology-based model specification languages. CompuCell3D is an open-source simulation platform that allows tissue-scale models to integrate easily with existing physiologically based pharmacokinetic models, with subcellular models of biological networks and with artificial intelligence (AI) [66]. However, CompuCell3D specifies models using a custom XML format (CC3DML) and Python scripts, thereby limiting model portability. While recent versions of CompuCell3D adopt a modular software architecture API, they currently lack integrated backing for model annotation, parameter constraints, provenance and validation data integration as part of model specification. This is also the case for other similar tools such as PhysiCell [67], Chaste [68] and CellSys [69]. An effort to promote best practices regarding model development and annotation workflows across multiple virtual-tissue platforms will be essential to making virtual-tissue models truly reusable and shareable.

## Use of scorecards and simple rules to enhance model reproducibility

The reproducibility of scientific results is of utmost importance in research. The team developing the BioModels database of mathematical models of biological systems [2] has attempted to systematically reproduce 455 kinetic models published in peer-reviewed research articles from 152 journals [70]. Unfortunately, about half (49%) of the models could not be reproduced using the information provided in the published manuscripts. With further effort, an additional 12% of the models could be reproduced either by empirical correction or feedback from authors. The main reasons why reproduction failed were missing parameter values, followed by missing initial conditions and inconsistencies in the model structure. As a lightweight approach to improve reproducibility in systems biology, an eight-point scorecard is proposed to be used by authors, reviewers and editors, highlighting critical manuscript features that assist reproducibility [70]. This effort could be extended to any model repository to ensure the reproducibility of published results.

Along the same lines, a recent publication suggests 10 simple rules for credible modelling practices in healthcare [71]. These rules include (i) a clear definition of the modelling context and scope, (ii) the use of relevant data, (iii) model evaluation within the biological context, (iv) explicit mention of model and simulation limitations, (v) the use of version control, (vi) appropriate and detailed documentation, (vii) broad dissemination of the modelling results, (viii) external review of the content, (ix) testing of competing implementations where possible and finally, (x) use of standards.

Table 1 summarises the main reasons for irreproducibility in computational systems biology and possible solutions.

**Table 1:** Main reasons for irreproducibility and possible solutions

**Main reasons for irreproducibility**	**Possible solutions**
Lack of parameter values	• Associate SED-ML files to the model and use a COMBINE archive to group all files • Where SED-ML files are not possible, provide tables with parameters and proper annotations of their sources • Use MIRIAM annotations and proper identifiers to reference sources • Use harmonised identifiers for the model entities, such as HUGO Gene Nomenclature Committee (HGNC), UniProt IDs or GeneSymbols • Provide code scripts in open-access repositories such as GitLab or GitHub and a clean, comprehensive read.me file • Prove a thorough list of tools, platforms, packages and dependencies used to build, analyse and simulate the model • Use standard formats for model files to ensure interoperability and avoid future issues with outdated software • Include a step-by-step methodology description in the Methods sections or Supplementary materials
Lack of initial conditions for simulations	
Inconsistencies in model structure, such as missing interactions	
Lack of comprehensive description of the system	
Lack of proper annotations for every interaction	
Inconsistencies in the naming of model entities	
Inconsistent description of experiments	
Outdated software	
Missing parts in the description of methodology	
Missing scripts in the code for model experiments	

## Discussion

### General remarks and fundamental questions

More tools and platforms must incorporate standards to enable their implementation by the modellers. Communities should also reach a consensus regarding whether annotations should be included as part of the model file or offered in a separate file as advocated in [9]. This separate file could be combined with the model and the simulation settings using a COMBINE archive [72]. There are limitations in existing ontologies for describing mechanistic information and multicellular phenotypes. The cell behaviour ontology initiative tried to develop semantic description of multicellular computational models, but it requires much more coordinated effort and development [73].

In general, model construction starts from the literature and prior knowledge, often in the form of an existing computational model that was probably built to address different questions or in a different biological context. First, the modeller compiles a list of components from relevant experiments and disciplinary knowledge/data. Subsequently, the modeller performs simulations to train and validate the model based on reference data. Model building is iterative and requires progressive model refinement to reach a robust model, which can be used for designing experiments and hypothesis testing. To maximize a model’s impact, its representation and distribution must support its reproducibility, accessibility and reusability. While these aims are independent of the computational methodology employed, each has specific needs for its realisation.

One fundamental question regarding models and their minimum associated information is what constitutes a model? For example, is a model a network or a set of rules/equations? Furthermore, when a model is published, how should it be accompanied by metadata including the initial conditions, simulation settings and annotation of the model entities to ensure maximum reproducibility and reusability? These questions need to be addressed every time standard practices are defined and proposed.

In this section, we describe experiences with use cases and applications from several collective efforts that apply and harmonise best practices.

### Recent collective efforts that promote best practices and enhance communication across communities

Since 2019, collective efforts have emerged to tackle different pandemic challenges. The COVID-19 Disease Map project [74] brought together scientists with various backgrounds to build a computational repository of virus-host interaction mechanisms. This large-scale international effort fostered exchanges and collaboration across disciplines. The COVID 19 Disease Map community adopted the SBGN standards for graphical models [6] and defined the minimal annotation information required for such diagrams. The aim was to use the map-to-model framework [15] to create automatically annotated executable Boolean models, as suggested in [1]. The project also sparked interesting questions about tools and platforms and highlighted the need to create interoperable pipelines to bridge static and dynamic representations of disease mechanisms.

In parallel, the logical modelling community proposed an adaptation of SED-ML to cover the needs of logical models. The community also incorporated more tools into CoLoMoTo notebooks [56] to promote reproducibility and interoperability. An adapted SED-ML for logic-based models would promote the use of COMBINE archives, harmonising best practices of model development across communities.

Computational biology models should also follow the FAIR principles. Most of the good-practice guidelines followed and suggested by individual systems biology communities fulfil the FAIR criteria, even when not explicitly mentioning the FAIR facets. For example, FAIR research objects should be findable [42]. The BioModels database, an open repository for simulation studies of biomedical systems [2], provides each model with a persistent Identifier (ID) that can be represented as a uniform resource identifier to guarantee global uniqueness [75]. The persistent identifier allows a specific model to be referenced from outside the repository, for example, when reusing the model code in a simulation.

Models need to meet the accessibility criteria in FAIR [42]. In addition, one would need to state precisely how a model can be reused to employ such a model for clinical biomedical tasks. For example, model code and associated metadata should be retrievable using standardised communication protocols like HTTPS or SPARQL. Finally, automated download options for models are as important as easy access to model code for scientists accessing the front end of a model repository. Biomodels, for example, allows for the download of model code in both original model formats and SBML [7], using simple HTTPS. An alternative is programmatic access to the model collection via REST interfaces.

Computational biology models have long been reused and provide many examples for adopting FAIR principles. For example, the HealthEcco project (https://healthecco.org/) integrates health-related data for several diseases in a graph database at the intersection between health and systems medicine. HealthEcco relies solely on standardised interfaces to access data from open repositories. For their data section on COVID-19, HealthEcco integrated computational biology models from the COVID collection of Biomodels (https://www.ebi.ac.uk/biomodels/covid-19) with patents, biomedical ontologies, PubMed entries and clinical trials [76].

More recently, a workgroup at COMBINE 2021 (https://combine-org.github.io/events/) investigated the FAIR metrics for computational models to determine how FAIRification can lead to higher quality, more reliability and, ultimately, more frequent reuse of model-based results in biomedical applications.

### What are the challenges to overcome as separate communities and collectively?

As already mentioned, most current simulation environments and tools handle one or two methodologies per scale. The use of standard methodology-independent formats for model specification could improve interoperability and help standardize the description of spatial phenomena in cross-scale integration languages. Tools and platforms which support standards are of utmost importance. Annotations and references are indispensable for assessing a model’s quality and facilitating its reusability, and both should be independent of the modelling methodology. They could also serve as minimum quality features and prerequisites for publishing computational models. The description of model structure, including components and reactions with proper references to sources and simulation settings, could be embedded in all publications to ensure transparency. Many scientific journals have updated their policies to include hosting the code, datasets and resources necessary to reproduce the research detailed in submitted manuscripts. It would be helpful if this support could extend to computational models and simulations, as very often published models are not annotated at all, include no references to the sources used to infer reactions and the simulation settings are vaguely described, hampering the reproducibility of the results and the reusability of the models. Support for and use of public, shared repositories can improve the findability and reusability of models. As these issues affect all computational models, modelling communities should improve communication to collectively develop solutions to these challenges.

### A tentative framework to improve the comprehensiveness, accessibility, reusability, interoperability and reproducibility of computational models in biology

**Figure 3:**
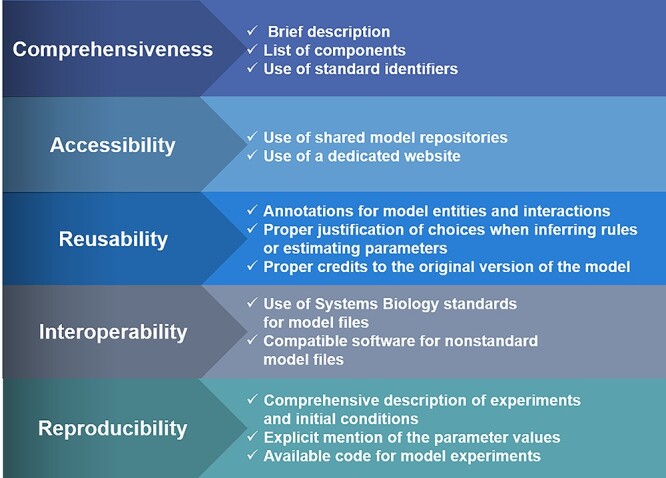
Main challenges to increase the impact of computational models in biology and tentative suggestions to address them.

In this section, we propose a tentative guide and a checklist that could be useful to assess a model’s impact in terms of comprehensiveness, accessibility, reusability, interoperability and reproducibility (Figure 3). While preliminary, the checklist can provide the basis for good practices aligned with systems-biology community efforts for both modellers and reviewers when evaluating a computational model prior to or during peer review (Table 2).

**Table 2:** A tentative checklist that could be used by both modellers and reviewers to assess a model’s compliance with systems biology communities guidelines

**Core aspects**	**Check points**	**Proposed actions**
***Comprehensiveness***	• Brief description of the biological system and mechanisms to model	• Simple text file
	• Consistency between the model structure and the described biological mechanism	• Abstract figure, textbook illustration, text description
	• List of components and interactions along with proper annotations	• Use of standard identifiers such as PubMed IDs
***Accessibility***	• Use of shared model repositories	• BioModels, other dedicated repositories
	• Use of a dedicated website besides supplementary materials	• Provide URL of the repository (*e.g.*: GitHub, GitLab)
***Reusability***	• Zip files of the model documentation	• COMBINE archive • FAIR principles
	• Standard annotations for model entities and interactions	• MIRIAM guidelines, use of unified identifiers (UniProt IDs, HGNC symbols, *etc*.)
	• Description of equations or rules used in the model	• Explicitly mentioned in the Methods section, in the Supplementary files, or in a dedicated accompanying webpage (e.g. GitHub)
	• Proper justification of choices when inferring rules or estimating parameters	• Explicitly mentioned in the Methods section or in the Supplementary files
	• Proper credits to the original version of the model	• Mentioned in the article and encoded in the model file with the use of proper identifiers
***Interoperability***	• Use of Systems Biology standards for model files	• Provide model files in standardised formats
	• Non-standard format of model files	• List of compatible software for model analysis
***Reproducibility***	• Comprehensive description of experiments and initial conditions	• SED-ML • COMBINE archive
	• Explicit mention of parameter values	• Tables with parameters and proper annotation of their sources
	• Available code for model experiments	• Scripts in open-access repositories such as GitLab or GitHub
	• Detailed description of methodology	• Step-by-step methodology description in the Methods section or Supplementary materials

#### Model comprehensiveness

Provide a brief description (half a page) of the biological system and mechanisms encoded in the model. The model structure must be consistent with the biological processes it aims to describe. Give a list of all the components and interactions along with proper annotations such as PubMed IDs, KEGG IDs, Reactome IDs, *etc*. that justify their inclusion in the model. Use the minimal information required in the annotation of models (MIRIAM, https://co.mbine.org/standards/miriam) guidelines for the consistent annotation and curation of computational models in biology.

#### Model accessibility

Use shared model repositories, like BioModels, to ensure that the model is findable by a larger audience, and also repositories specific to tools and/or platforms, such as GINsim or CellCollective model repositories, to reach community members.

#### Model reusability

Provide proper documentation of the equations or rules used in the model. Make sure to mention and justify the choices made when inferring rules or estimating parameters. Use consistent and comprehensive naming of model entities with standard identifiers to avoid ambiguities. The use of MIRIAM guidelines is highly recommended. Provide proper credits to the original version of the model, if you add modifications. FAIR principles for data stewardship [19] are also recommended to maximize the model’s reusability. Where possible, provide zip files of the model documentation following COMBINE archive guidelines (http://co.mbine.org/standards/omex).

#### Model interoperability

Use Systems Biology standards for model building, annotation and simulation and provide files in standard formats, where possible. Otherwise, provide details on the software and platforms used to analyse the model.

#### Model reproducibility

Provide a brief description of *in silico* experiments along with detailed initial conditions such as initial concentrations or states, updating schemes, time frame and number of replicas for all simulation scenarios tested. Give all sets of equations and details about parameter values or ranges used. In the case of logic-based models, provide the logical formulae. Be transparent about possible limitations and cases that the model failed to reproduce. Make sure the naming of all model entities is consistent between model description and model file. The authors should also consider adopting the scorecards proposed in [70].

## Outcomes and outlook

Frequent communication and exchanges between various Systems Biology communities create the optimal conditions for establishing a common and consensual framework for annotated, accessible, reproducible and interoperable computational models in biology. Regular community meetings are essential to evaluate efforts, exchange experiences and address the challenges of computational systems biology in a collective and community-driven spirit. There is a pressing need for a harmonised and easily applicable framework that would improve computational models’ accessibility, reusability, interoperability and reproducibility. To achieve this goal, we propose here a tentative framework that could be adopted by the different systems biology communities, and also by editors and reviewers, as an important step before scientific publication. Our aim is not to enforce a one-size-fits-all solution but to create a framework that is flexible and adaptable to accommodate the particular needs of various modelling approaches in computational systems biology.

**Key Points**

- The systematic use of standards minimises model ambiguities and enhances models’ reusability and tools’ interoperability.
- FAIR principles significantly improve the quality of modelling pipelines.
- Model annotations, references and specifications of detailed simulation settings are required to ensure transparency during peer-review and publication.
- Specialised best practice workflows and description standards need to be developed to address inter-scale connections in multiscale models.
- The efforts of different communities should be coordinated and aligned to accelerate progress and avoid duplicate efforts.

## Author Biographies

**Anna Niarakis** is an Associate Professor at Univ Evry, University of Paris-Saclay. Her research focuses on applying computational systems biology approaches such as network integration and dynamical modelling to decipher complex human disease mechanisms.

**Dagmar Waltemath** is a Professor of medical informatics at the University of Medicine, Greifswald. She is the chair of the Computational Modelling in Biology Network and an advocate of the findability, accessibility, interoperability and reusability (FAIR) principles and open standards for modelling and simulation in biomedical science.

**James A. Glazier** is a Professor of Intelligent Systems Engineering, an Adjunct Professor of Physics and Director of the Biocomplexity Institute at Indiana University, Bloomington, USA. His research focuses on developing and applying multiscale modelling and model shareability tools.

**Falk Schreiber** is a Professor at the University of Konstanz and an Adjunct Professor at Monash University. His research focuses on analysing the structure and dynamics of biological networks, the modelling of biological processes and the interactive visualization and immersive analytics of life science data.

**Sarah Keating** is a Senior Research Software Developer at University College London (UCL) and a coordinator of the Computational Modelling in Biology Network initiative. Her research focuses on standard formats for model exchange and reuse. She has been the lead developer of libSBML and other infrastructure supporting standards for more than 15 years.

**David Nickerson** is a Senior Research Fellow at the Auckland Bioengineering Institute. He is the vice-chair of the Computational Modelling in Biology Network and an investigator at the Center for Reproducible Biomedical modelling. His research focuses on physiological model development standards and tools for reproducibility and reuse.

**Claudine Chaouiya** is an Associate Professor at I2M, Aix-Marseille University. Her research focuses on developing appropriate methods and tools for analysing large logical models of regulatory and signalling networks revolving around cell fate decisions in single-cell and multicellular contexts.

**Anne Siegel** has been a research director at Centre National de la Recherche Scientifique (CNRS) since 2010 and works at the IRISA lab in the Bioinformatics Dyliss Group. Her research activities focus on interfaces between computer science and biology. She develops symbolic knowledge representation and integration methods to analyse large-scale biological systems and microbial communities, especially at the metabolic scale.

**Vincent Noël** is a researcher at the Institut Curie. His research interests are the modelling of biological systems, from constructing and analysing models to optimising their simulations.

**Henning Hermjakob** leads the Molecular Systems services at European Molecular Biology Laboratory -European Bioinformatics Institute (EMBL-EBI), which provide worldwide reference data resources in interactomics (IntAct), pathways (Reactome) and systems biology models (BioModels). He received his MSc in Bioinformatics from the University of Bielefeld and has been at EMBL-EBI since 1997.

**Tomáš Helikar** is an Associate Professor in the Department of Biochemistry at the University of Nebraska-Lincoln. His research focuses on using integrative modelling pipelines to elucidate how aberrant changes in biological networks result in disease to develop more effective therapies strategically.

**Sylvain Soliman** is a researcher at Inria, Saclay. His research interests focus on computational biology and theoretical computer science. He has been one of the leading developers and maintainers of the BIOCHAM modelling and analysis platform for more than 10 years.

**Laurence Calzone** is a researcher at the Institut Curie. Her research activities revolve around building knowledge maps, constructing logical models and developing tools for improving and optimising qualitative models and their predictions.
