# Correction to: Addressing *barriers in comprehensiveness, accessibility, reusability, interoperability and reproducibility of computational models in systems biology*

**DOI:** 10.1093/bib/bbae253

**Published:** 2024-05-12

This is a correction to: Anna Niarakis, Dagmar Waltemath, James Glazier, Falk Schreiber, Sarah M Keating, David Nickerson, Claudine Chaouiya, Anne Siegel, Vincent Noël, Henning Hermjakob, Tomáš Helikar, Sylvain Soliman, Laurence Calzone, Addressing barriers in comprehensiveness, accessibility, reusability, interoperability and reproducibility of computational models in systems biology, *Briefings in Bioinformatics*, Volume 23, Issue 4, July 2022, bbac212, https://doi.org/10.1093/bib/bbac212.

This article has been corrected to update the National Institute for Biomedical Imaging and Bioengineering award grant number to D.N. from P41GM109824 to P41EB023912.

